# Management of vertebral compression fracture in general practice: BEACH program

**DOI:** 10.1371/journal.pone.0176351

**Published:** 2017-05-04

**Authors:** Rodrigo Z. Megale, Allan Pollack, Helena Britt, Jane Latimer, Vasi Naganathan, Andrew J. McLachlan, Manuela L. Ferreira

**Affiliations:** 1 Institute of Bone and Joint Research, The Kolling Institute, Sydney Medical School, The University of Sydney, Sydney, Australia; 2 School of Public Health, Family Medicine Research Centre, The University of Sydney, Sydney, Australia; 3 School of Public Health, The University of Sydney, Sydney, Australia; 4 The George Institute for Global Health, Sydney Medical School, The University of Sydney, Stdney, Australia; 5 Centre for Education and Research on Ageing, Concord Clinical School, The University of Sydney, Sydney, Australia; 6 Faculty of Pharmacy, The University of Sydney, Sydney, Australia; University of Toronto, CANADA

## Abstract

**Importance:**

The pain associated with vertebral compression fractures can cause significant loss of function and quality of life for older adults. Despite this, there is little consensus on how best to manage this condition.

**Objective:**

To describe usual care provided by general practitioners (GPs) in Australia for the management of vertebral compression fractures.

**Design, setting and participants:**

Data from the Bettering the Evaluation And Care of Health (BEACH) program collected between April 2005 and March 2015 was used for this study. Each year, a random sample of approximately 1,000 GPs each recorded information on 100 consecutive encounters. We selected those encounters at which vertebral compression fracture was managed. Analyses of management options were limited to encounters with patients aged 50 years or over.

**Main outcome(s) and measure(s):**

i) patient demographics; ii) diagnoses/problems managed; iii) the management provided for vertebral compression fracture during the encounter. Robust 95% confidence intervals, adjusted for the cluster survey design, were used to assess significant differences between group means.

**Results:**

Vertebral compression fractures were managed in 211 (0.022%; 95% CI: 0.018–0.025) of the 977,300 BEACH encounters recorded April 2005– March 2015. That provides a national annual estimate of 26,000 (95% CI: 22,000–29,000) encounters at which vertebral fractures were managed. At encounters with patients aged 50 years or over (those at higher risk of primary osteoporosis), prescription of analgesics was the most common management action, particularly opioids analgesics (47.1 per 100 vertebral fractures; 95% CI: 38.4–55.7). Prescriptions of paracetamol (8.2; 95% CI: 4–12.4) or non-steroidal anti-inflammatory drugs (4.1; 95% CI: 1.1–7.1) were less frequent. Non-pharmacological treatment was provided at a rate of 22.4 per 100 vertebral fractures (95% CI: 14.6–30.1). At least one referral (to hospital, specialist, allied health care or other) was given for 12.3 per 100 vertebral fractures (95% CI: 7.8–16.8).

**Conclusions and relevance:**

The prescription of oral opioid analgesics remains the common general practice approach for vertebral compression fractures management, despite the lack of evidence to support this. Clinical trials addressing management of these fractures are urgently needed to improve the quality of care patients receive.

## Introduction

Vertebral compression fractures (VCFs) are of increasing public health concern due to a rising prevalence in an ageing Australian population. Around 726,000 Australians are at risk of developing an osteoporotic vertebral fracture every year [1], and in fact, one in four women aged 80 years or over will have sustained one or more vertebral fractures [2, 3]. In general practice, the burden of this condition is likely underestimated as only around one third of vertebral fractures will be clinically diagnosed [4]. Even in the acute phase, most cases are not recognised at the time of their occurrence [5].

Acute pain is a common clinical presentation of symptomatic VCFs. Recent observational studies have shown that patients may report moderate to severe pain intensity after a VCF, with an average score of 7 on a 10-point visual analogue pain scale [6, 7]. Further, some patients may develop persistent pain with reduced function and quality of life [8–13].

Significant attention is currently paid to the secondary prevention of vertebral and non-vertebral fractures given that the presence of a VCF is an important predictor of future osteoporotic fractures [14]. However, pain relief is as important as osteoporosis treatment in older adults, because each additional day of immobility due to pain will result in further loss of muscle mass, strength, and functional capacity [15, 16]. Effective pain management may prevent prolonged bed rest, deconditioning and further losses of physiological and functional capacity, especially important among frail older adults. Unfortunately, there is no consensus on the clinical pathway for pain management in patients with VCF. The available guidelines differ markedly in their recommendations [17–20] and the scientific evidence on the effective management of VCF is scarce [21].

The lack of consensus in VCF management means that clinicians must rely on their own expertise when managing patients with symptomatic VCF, resulting in significant variation in usual care. Descriptive studies reporting such variation would provide valuable information to be used in public health planning. In Australia, general practitioners (GPs) are usually the first port of call and those who first manage VCF in outpatient settings. The aim of this study is to describe the usual management of VCF in older adults at consultations in Australian general practice and to identify gaps to be addressed in future research to inform best practice.

## Methods

### Population and settings

We analysed data from the Bettering Evaluation and Care of Health (BEACH) program, collected April 2005 to March 2015 inclusive. The data collection methods are described in detail elsewhere [22, 23]. In summary, the BEACH program is a continuous, national cross-sectional study of general practice activity in Australia. Each year, an ever-changing random sample of approximately 1,000 GPs each records details of 100 consecutive encounters with consenting patients (total approximately 100,000 encounters/year) on structured paper encounter forms. GPs are randomly selected from a national list of active GPs, defined as those for whom at least 375 GP services were claimed for Government rebates in the previous quarter. Patient reasons for the encounter (up to three), problems managed (which includes evaluated, treated or otherwise dealt with) (up to four), and treatments (linked by the GP) to each problem, are recorded as free text. The status of each problem–new (first presentation to a medical practitioner), or follow-up (previously managed problem)–was also indicated. Completed forms are returned to the research team, centrally coded in an Australian general practice interface terminology ICPC-2 PLUS [24], classified according to the International Classification of Primary Care, Version 2 (ICPC-2) [25]. Pharmaceutical were classified at generic level according to the World Health Organization’s (WHO) Anatomic Therapeutic Chemical (ATC) classification [26].

In this study, we used data from encounters at which VCFs were managed. VCF problems were defined as ICPC-2 PLUS code L84019 (“Fracture; compression (of); spine”). Analyses of management actions for VCF were limited to encounters with patients aged 50 years or over. This age group is considered to be at higher risk of VCF consequent to primary osteoporosis, than patients aged less than 50 years, among whom VCF is more likely to be associated with secondary osteoporosis or with major trauma [27].

The data elements used in this study of VCF management were: i) patient demographics; ii) co-morbidities managed iii) the management provided for during the consultation (medications prescribed or supplied by the GP and their prescribed daily dose; clinical treatments such as general and specific advice, counselling or education; procedural treatments including therapeutic actions and diagnostic procedures undertaken at the encounter; referrals to specialists, and to allied health services; and orders for pathology and imaging tests). Opioid analgesics included in the 5-digit ATC code as N02AA (except codeine and dihydrocodeine), N02AE or N02AB were considered “strong” opioids. Codeine (R05DA04 or N02AA59), dihydrocodeine (N02AA08) or opioid analgesics included in the 5-digit ATC code as N02AC and N02AX were considered “weak” opioids. The Australian and New Zealand College of Anaesthetists (ANZCA) opioid conversion table [28] was used to convert the daily opioid analgesic dose to morphine equivalents.

Antidepressants (N06AA, N06AB and N06AX), antiepileptics (N03AE, N03AF, N03AG, N03AX), anxiolytics (N05BA) or glucocorticoids (H02AB) were considered adjuvant pain medications when used in VCF management. In this analysis bisphosphonates (M05BA), combinations of bisphosphonates with other compounds (M05BB), strontium ranelate or denosumab (M05BX) were pooled under the label “anti-osteoporotic medication”. Non-pharmacological management approaches included clinical treatments involving general and specific advice, counselling or education, administrative processes and procedural treatments involving physical medicine/rehabilitation.

In Australia, there is a universal medical insurance scheme (Medicare Australia), which covers all or part of an individual’s cost for a GP visit. The national annual number of encounters at which VCF was managed was therefore estimated as the proportion of BEACH encounters at which VCF was managed multiplied by the national average annual number of GP consultation items claimed from Medicare over the period 2005–15.

### Statistical analysis

Descriptive analyses are presented as frequencies and mean rates. Using SAS^®^ 9.3, robust 95% confidence intervals (CI), adjusted for the cluster survey design are reported, except if less than three observations. Differences between group means were regarded as significant when 95% CIs did not overlap.

Results estimating the caseload of VCFs are reported as management rates per 100 GP encounters. As more than one problem could be managed at each encounter, management actions (such as medication prescription) are only those linked by the GP to the VCF problem and are reported as rates per 100 VCF problems managed.

The BEACH program is approved by the Human Research Ethics Committee of the University of Sydney and the Ethics Committee of the Australian Institute of Health and Welfare (project number 2012/130).

## Results

In this sample, for all age groups, 211 VCF problems were managed at 211 (0.022%; 95% CI: 0.018–0.025) of the 977,300 encounters recorded from April 2005– March 2015. These data were extrapolated to an estimated national annual average of 26,000 (95% CI: 22,000–29,000) encounters at which VCFs were managed by GPs.

### Description of encounters at which VCFs were managed

The majority of patients at VCF encounters were: female (65.1%); 65 years and over (64.8%); previously seen at the recording GP’s practice (95.7%). Of 211 VCF encounters, 186 (86.7%) were claimable from Medicare, and, of these, 85.2% were surgery consultations, whereas home, hospital or residential aged care visits accounted for 8.5%. Follow-up management of previously diagnosed VCFs (59.2%; 95% CI: 52.4–66.1) was more frequent than management of new cases (40.8%; 95% CI: 33.9–47.6) (Table 1).

**Table 1:** Main characteristics of encounters at which vertebral compression fractures (VCFs) were managed–BEACH, 2005–2015, all patient ages.

	**N (N = 211 VCF encounters)**	**Rate per 100 VCF^a^-encounters (95% CI^b^)**
**Encounters characteristics^c^**		
Female	136	65.1 (58.3–71.8)
Male	73	34.9 (28.2–41.7)
Age <50 years	40	19.0 (13.3–24.8)
Age > = 50 years	170	81.0 (75.2–86.7)
Age > = 65 years	136	64.8 (57.9–71.7)
Patient new to practice	9	4.3 (1.5–7.1)
Patient seen previously	200	95.7 (92.9–98.5)
Surgery consultations	161	76.3 (70.3–82.3)
Hospital, residential aged care and home visits	16	7.6 (3.8–11.3)
**VCF management**	**N (N = 211 VCF problems)**	**Rate per 100 VCF^a^ problems (95% CI^b^)**
First presentation of VCFs	86	40.8 (33.9–47.6)
Follow-up of previously assessed VCFs	125	59.2 (52.4–66.1)
Additional investigation		
At least 1 imaging exam	67	31.8 (25.2–38.3)
At least 1 pathology exam	10	4.7 (1.8–7.6)
At least 1 medication prescribed	128	60.7 (54.1–67.2)
At least 1 referral	26	12.3 (4.2–22.3)
Hospital	5	2.4 (0.3–4.4)
Specialist	12	5.7 (2.5–8.8)
Allied health services	8	3.8 (1.2–6.4)
Other referrals	1	0.6*

a) Vertebral compression fracture.

b) Confidence interval.

c) N missing: sex 2; age 1; patient new to practice 2.

^*^ 95% CI not reported for n<3.

Additional investigations were ordered for less than half of the VCF problems managed. At least one imaging test was ordered for 31.8% (25.2–38.3) and at least one pathology test for 4.7% (1.8–7.6) of VCF problems. Pharmacological treatment was the most common management action for VCF, at least one medication being prescribed, supplied or advised for 60.7% (54.1–67.2) of VCF problems managed. At least one referral (to hospital, specialist, allied health care or other) was given for 12.3% (7.8–16.8) (Table 1).

### Patient’s reason for encounter and other problems managed

Patients described 331 (156.9 per 100 VCF encounters) reasons for encounter (RFEs). Classified by ICPC-2 chapter, musculoskeletal complaints (n = 145) were the most common (68.7 per 100 VCF encounters), representing 43.8% of all RFEs. Back complaint (48.3 per 100 VCF encounters), trauma/injury (not otherwise specified) (6.2) and fracture (5.7) were the top 3 musculoskeletal RFEs.

On average, 75.4 (62.2–88.5) problems (other than VCF) were managed per 100 VCF encounters, most commonly being circulatory (13.3 (7.7–18.9) per 100 VCF encounters), musculoskeletal (12.8 (7.8–17.8)) and psychological (9.5 (5.3–13.7)). Hypertension (4.3 per 100 VCF encounters), lipid disorders (3.8), osteoporosis (3.8), sleep disturbance (3.3) and depression (2.4) were the top 5 individual problems co-managed in VCF encounters (Table 2).

**Table 2:** Reasons for encounter (RFEs) and other problems managed at vertebral compression fracture (VCF) encounters–BEACH, 2005–2015, all patient ages.

	**Number of RFEs**	**Rate per 100 VCF^a^-encounters (95% CI^b^) N = 211**
**All RFEs^c^ recorded**	331	156.9 (146.3–167.5)
**RFEs from ICPC-2^d^ musculoskeletal (MSK) chapter**	145	68.7 (61.4–76.0)
**Top 3 RFEs from ICPC-2 MSK^e^ chapter**		
Back complaints	102	48.3 (41.3–55.3)
Trauma / injury NOS^f^	13	6.2 (2.6–9.7)
Fracture	12	5.7 (2.5–8.8)
**RFEs from other (non-MSK) ICPC-2 chapters**	186	88.2 (76.4–99.9)
Top 3 RFEs from other ICPC-2 chapters		
Test results	46	21.8 (15.1–28.5)
Prescriptions	29	13.7 (8.8–18.7)
General check-up	5	2.4 (0.3–4.4)
	**Number of other problems managed**	**Rate per 100 VCF-encounters (95% CI) N = 211**
**Problems (other than VCF) managed**	159	75.4 (62.2–88.5)
Hypertension	9	4.3 (1.5–7.0)
Lipid disorders	8	3.8 (1.2–6.4)
Osteoporosis	8	3.8 (1.2–6.4)
Sleep disturbance	7	3.3 (0.9–5.8)
Depression	5	2.4 (0.3–4.4)

^a)^ Vertebral compression fracture.

^b)^ Confidence interval.

^c)^ Reasons for encounter

^d)^ International Classification for Primary Care-2

^e)^ Musculoskeletal

^f)^ Not otherwise specified.

Of VCF encounters, 170 (80.6%) were with patients aged 50 years or over (those at higher risk of primary osteoporosis). In this group the proportions of women, patients previously seen by GP, and first presentation of VCFs; the likelihood of additional investigations, pharmacological treatment and referrals did not significantly differ from those of the total sample. Likewise, the rates of the top 3 musculoskeletal RFEs and the top 5 other problems managed were similar to the rates presented for the whole sample (data not shown).

### Pharmacological and non-pharmacological VCF treatment at encounters with patients aged 50 years or over

At VCF encounters with patients aged 50 years or over, opioids were the top analgesic class prescribed for VCF (47.1 per 100 problems; 95% CI: 38.4–55.7) (Table 3). For new cases of VCF weak opioid analgesics (20.3; 95% CI: 10.1–30.4) were more often prescribed than strong opioids (12.7; 95% CI: 5.3–20.0). In contrast at follow-up encounters prescriptions for strong opioids analgesics were more common than for weak opioids (47.3; 95% CI: 35.1–59.4 vs. 12.1; 95% CI: 5.5–18.7). The prescription of paracetamol (8.2) was less frequent than opioids for all VCF problems. No significant difference was found in the paracetamol prescription rate for new cases (10.1; 95% CI: 3.4–16.9) and previously diagnosed VCF cases (6.6; 95% CI: 1.4–11.8). Non-steroidal anti-inflammatory drugs (NSAIDs) were less frequently prescribed (4.1; 95% CI: 1.1–7.1) and adjuvant pain medication prescriptions were rare.

**Table 3:** Pharmacological and non-pharmacological treatment for new (first presentation) vertebral compression fracture (VCF) problems and for previously-assessed VCF problems–BEACH, 2005–2015, patients aged 50 years and over.

	First presentation of VCF (new problem) (N = 79)		Follow-up of VCF (previously-assessed problem) (N = 91)		All VCF problems (N = 170)	
Pharmacological treatment	Number of medications or treatments	Rate per 100 VCF^a^ problems (95% CI^b^)	Number of medications or treatments	Rate per 100 VCF problems (95% CI)	Number of medications or treatments	Rate per 100 VCF problems (95% CI)
**Medications prescribed**	54	68.4 (48.9–87.8)	78	85.7 (67.6–103.9)	132	77.6 (64.6–90.7)
**Opioid analgesics**	26	32.9 (21.5–44.4)	54	59.3(47.1–71.6)	80	47.1 (38.4–55.7)
“Strong” opioid	10	12.7 (5.3–20.0)	43	47.3 (35.1–59.4)	53	31.2 (23.2–39.1)
“Weak” opioid	16	20.3 (10.1–30.4)	11	12.1 (5.5–18.7)	27	15.9 (10.0–21.8)
**Paracetamol**	8	10.1 (3.4–16.9)	6	6.6 (1.4–11.8)	14	8.2 (4.0–12.4)
**NSAIDs^c^**	2	2.5*	5	5.5 (0.7–10.3)	7	4.1 (1.1–7.1)
**Adjuvant pain medication**	0	0.0*	4	4.4 (0.1–8.7)	4	2.4 (0.0–4.7)
**Anti-osteoporotic medication**	5	5.5 (0.7–10.3)	12	15.2 (6.6–23.8)	17	10.0 (5.2–14.8)
**Non-pharmacological treatment**	16	20.3 (9.3–31.2)	22	24.2 (13.2–35.2)	38	22.4 (14.6–30.1)

^a)^ Vertebral compression fracture problems managed.

^b)^ Confidence interval.

^c)^ Non-steroidal anti-inflammatory drugs.

^*^ 95% CI not reported for n<3.

Anti-osteoporotic medications including bisphosphonates, strontium ranelate or denosumab, were prescribed in new cases at a rate of 5.5 (0.7–10.3) per 100 VCF problems, and in follow-up care at 15.2 (6.6–23.8) per 100. There were 22.4 (14.6–30.1) non-pharmacological treatments (counselling, advice, education or physical medicine/rehabilitation) per 100 VCF problems. (Table 3).

The mean and median daily dose of oral opioids analgesics prescribed for VCF pain management is shown in Table 4. Except for one prescription for 90 mg of morphine sulphate per day, opioid medications were prescribed in relatively low doses, with a mean daily dose ranging from 18 to 40 mg morphine equivalents.

**Table 4:** Mean daily dose of prescribed oral opioid analgesics for VCF-related pain in encounters with patients aged 50 years and over.

	Number of prescriptions	Opioid mean daily dose		Opioid median daily dose	
		mg	mg morphine equivalent	mg	mg morphine equivalent
Oxycodone	26	24	36	15	23
Oxycodone/ Naloxone	7	21	31	8	11
Tramadol	13	200	40	150	30
Paracetamol/ Codeine	6	170	22	210	35
Paracetamol/ Dextropropoxyphene	2	179	18	179	18
Morphine sulphate	1	90	90	90	90

## Discussion

This descriptive study provides important information about current management of VCF by GPs in Australia. This information was derived from data collected through the BEACH program, which has been previously shown to accurately reflect how GPs manage primary care conditions [23, 29].

While this study found that VCFs were managed at a rate of 2/1000 GP encounters, this figure does not reflect the incidence or prevalence of the condition in Australia, but rather the caseload of VCFs in general practice. In the absence of specific protocols, it is difficult to determine the real burden of this condition because: a) most VCFs are asymptomatic [30]; b) even symptomatic VCFs can be undiagnosed in older patients with acute low back pain [31, 32]; and c) diagnosed VCFs are frequently under-reported [33, 34]. Our study has shown that, on average, nationally, each year over the 10 years of this study, 26,000 (22,000–29,000) encounters in general practices involved management of VCF. This represents an average of approximately 70 (60–80) VCFs being managed every day in Australia.

At about 20% of VCF encounters patients were under 50 years of age and, therefore secondary osteoporosis or major trauma (burst fractures) was probably associated with some vertebral fractures. To assess a subsample in which primary osteoporosis was most likely to be a reason for the VCF, we separately analysed the data for encounters with patients aged 50 years or over. This cut-off age has been used in previous clinical trials addressing the treatment efficacy of VCF [13, 35–41]. However, we found, that management of VCFs is quite consistent across age groups, and no significant difference was observed when the whole sample was compared to the older subgroup in terms of likelihood of investigation, pharmacological and non-pharmacological treatment and referral.

In almost half of the VCF encounters, back complaint was one of the patients’ expressed reasons for seeking medical care, and prescription of analgesic drugs was the most frequent management action for the VCF. Interestingly, management of other problems was very common in VCF encounters (75.4 per 100 VCF encounters), probably reflecting the fact that most patients with vertebral fractures are older and have comorbidities. Although most guidelines are developed for patients with a single disease and rarely deal with comorbidities [42, 43], future guidelines on management of VCF should consider the number and types of comorbidities that may be present when recommendations are being developed.

The most important information extracted from this study concerns VCF management in the last decade. The BEACH program directly links management actions to the specific condition being managed and so it was possible to get accurate information on VCF management. Based on BEACH data, it seems that GPs focused their management on pain relief. Unfortunately, we currently lack robust evidence supporting a specific pharmacological treatment for VCF pain in older adults. In the past, the World Health Organisation pain ladder management [44] was commonly used for guiding pain relief treatment options, but concerns about the use of NSAIDs in older patients [45–48] is resulting in an increased use of opioids analgesics.

Our data suggest that Australian GPs are more likely to prescribe opioids analgesics in low doses rather than paracetamol or NSAIDs for VCF-related pain. This practice, supported in part by the American Geriatric Society (AGS) [49], comes at a high cost to the patient, given the well-known side effects associated with opioids, including constipation, nausea and vomiting, sedation, impaired judgment, impaired psychomotor function and respiratory depression [48, 50].

In addition, our findings raise concerns regarding a significant number of strong opioid analgesic prescriptions for patients at follow-up for their VCFs. This could suggest that patients are remaining on strong opioids after an acute VCF and also that the need for strong opioids did not decrease over time. The increasing prevalence of opioid analgesic use in Australia has been reported in previous studies [51, 52]. The rates of opioid analgesic prescription at VCF follow-up encounters in our study support this concern.

According to the AGS 2009 Panel on the Pharmacological Management of Persistent Pain in Older Persons, use of opioid analgesics is recommended for patients with moderate to severe pain, pain-related functional impairment or diminished quality of life because of pain [49]. However, the AGS recommendations are not evidence-based but based on the clinical experience and the consensus of panel members.

The use of opioid analgesics as first line therapy, common practice for pain management in our study, should be re-evaluated. Only two studies [53, 54] comparing the use of opioid analgesics with other analgesics or placebo were found in a recent systematic review addressing non-surgical treatment for VCF [21]. Of these, one had insufficient statistical power to enable comparative efficacy analyses due to the premature cessation of the study [53] and the second included only 7 participants in the opioid analgesic treatment group [54]. Although in both trials the groups receiving opioid analgesics had lower pain intensity than controls, immediate and short-term effects of opioid analgesics on pain were found inconsistent across trials with different comparators. Thus, there is very little evidence for the benefits of opioid analgesics in patients with pain due to VCF, and new high-quality trials are needed to address the best approach for this condition before opioid medication is recommended as first line therapy for VCF.

Interestingly, in only a few encounters were the patients referred to allied health professionals (3.8 per 100 VCF problems). Although the scientific evidence on the effectiveness of most non-pharmacological treatments in VCF is conflicting [21, 55–57], a multimodal approach, using both pharmacological and non-pharmacological treatments, is strongly recommended for pain treatment in older patients. Non-pharmacologic treatment including physiotherapy has considerably less frequent and less severe adverse events, and is central in improving pain, muscle strength, posture and mobility in these patients.

Our results have shown that anti-osteoporotic medication was prescribed for only 10% of the VCFs managed at the recorded encounters. This is a very low rate, however, we acknowledge it might not represent the total rate of prescription of osteoporosis treatments for patients with VCF, given we do not have access to medication already in use or prescribeb at follow-up encounters for VCFs for the sampled patients. Anyhow, this data raises suspicion that underdiagnosis and undertreatment of osteoporosis after a VCF might have been taking place in Australia.

Readers must be aware the diagnosis method used by GPs to come to the diagnosis of VCFs or the date of VCFs was not available in the BEACH program and therefore it is not possible to distinguish acute and chronic fractures in our dataset. The term “new fractures” refers to the first visit for a VCF in any one patient rather than acute fractures. There are also other limitations to our study. First, there might be inconsistencies in diagnostic coding, even considering that the coding of GP diagnoses of VCF was determined by trained coders using the ICPC-2 PLUS terminology. In addition, the BEACH program does not follow the patient over time; therefore we cannot identify changes in an individual’s VCF management. Finally, our data describes Australian general practice activity and may not reflect the clinical practice in other international settings.

Although patients with VCF might suffer from both nociceptive and neuropathic pain [58], which could play a role on analgesics prescription, our data have not provided details on the nature of pain for included encounters. Medication prescription in this study was done at the general practitioner’s own discretion. Providing recommendations for or against the use of specific analgesic approach is beyond the scope of this study. However, we are concerned that long-term prescription of opioid analgesics seems to be a common practice for VCF-related chronic pain whereas non-pharmacological approaches seem to be neglected.

## Conclusion

The caseload of vertebral compression fractures in primary care cannot be ignored. We estimate that in Australia around 70 GP encounters will take place every day to manage VCFs. The prescription of analgesic drugs, particularly oral opioid analgesics, is the most common management action despite the lack of evidence supporting this practice. Referrals to allied health professionals were rarely reported for VCF management. Although we lack evidence on what constitutes the best treatment for symptomatic VCF in older adults, it seems sensible to begin with treatments that may reduce pain and improve mobility without the risk of significant adverse side effects. This means that a greater use of allied health professionals to deliver a multimodal approach to pain may be preferable to the current long-term prescription of opioid analgesics.
