# Correction: Management of vertebral compression fracture in general practice: BEACH program

**DOI:** 10.1371/journal.pone.0193531

**Published:** 2018-02-22

**Authors:** Rodrigo Z. Megale, Allan Pollack, Helena Britt, Jane Latimer, Vasi Naganathan, Andrew J. McLachlan, Manuela L. Ferreira

There is an error in the first sentence of the second paragraph in the Discussion section. The correct sentence is: While this study found that VCFs were managed at a rate of 2/10,000 GP encounters, this figure does not reflect the incidence or prevalence of the condition in Australia, but rather the caseload of VCFs in general practice.
